# Clinical Neuropathology practice guide 3-2014: Combined nerve and muscle biopsy in the diagnostic work-up of neuropathy – the Bordeaux experience 

**DOI:** 10.5414/NP300740

**Published:** 2014-03-12

**Authors:** Anne Vital, Claude Vital

**Affiliations:** 1 University of Bordeaux, Institut des Maladies Neurodégénératives,; 2 CNRS, Institut des Maladies Neurodégénératives, and; 3 Pathology Department, Bordeaux University Hospital, Bordeaux, France

**Keywords:** amyloidosis, mitochondrial cytopathy, muscle and nerve biopsy, sarcoidosis, vasculitis

## Abstract

Simultaneous combined superficial peroneal nerve and peroneous brevis muscle biopsy, via the same cutaneous incision, allows examination of several tissue specimens and significantly improves the diagnosis of systemic diseases with peripheral nerve involvement. Vasculitides are certainly the most frequently diagnosed on neuro-muscular biopsies, but this procedure is also well advised to asses a diagnosis of sarcoidosis or amyloidosis. More occasionally, combined nerve and muscle biopsy may reveal an unpredicted diagnosis of cholesterol embolism, intra-vascular lymphoma, or enables complementary diagnosis investigations on mitochondrial cytopathy or storage disease.

## Introduction 

Several books were devoted to pathology on nerve biopsy [1, 2, 3, 4, 5, 6] and were followed by several papers [7, 8, 9, 10]. Simultaneous combined superficial peroneal nerve and peroneous brevis muscle biopsy may be chosen on purpose because it allows examination of several specimens and significantly improves the diagnosis of systemic diseases with peripheral nerve involvement. We describe the surgical and technical procedures, and revisit our experience on nerve and muscle specimens removed via a single incision. A literature review reminds that several authors favored availability of muscle specimens to improve diagnosis performances particularly in vasculitis, sarcoidosis, amyloidosis, and more occasionally, cholesterol embolism, intra-vascular lymphoma, mitochondrial disorders, or storage diseases. 

## Materials and methods 

**Figure 1:**
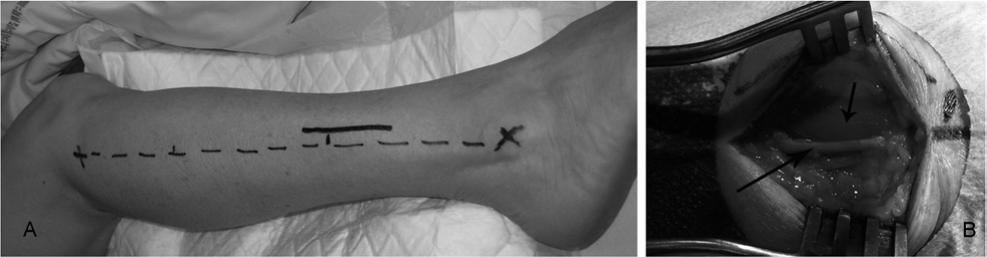
Surgical procedure for combined nerve and muscle biopsy: an incision has to be performed on the lower third of the leg, 1 cm anterior to a line joining the fibular apex to the lateral malleous at the ankle (A); the superficial peroneal nerve (long arrow) and the peroneous brevis muscle (short arrow) are well visible (B).

Combined nerve and muscle biopsy requires an appropriate surgical area, competent staff, and aseptic conditions. The aim is to biopsy the superficial peroneal nerve, and the peroneous brevis muscle via a single skin incision on the antero-lateral surface of the leg. Under local anesthesia, a 5 cm incision is performed on the lower third of the leg, 1 cm anterior to a line joining the fibular apex to the lateral malleolus at the ankle (Figure 1A). The superficial peroneal nerve and the peroneous brevis muscle are clearly visible (Figure 1B). After a proximal section of the superficial peroneal nerve, a 3-cm specimen is gently lifted without stretching or crushing and then removed by distal section. This specimen is divided into three pieces; one is immersed in buffered formalin for light microscopy, another in buffered glutaraldehyde for electron microscopy, and the third is frozen mainly for immunofluorescence. The peroneous brevis muscle biopsy is fixed for routine light microscopy, for electron microscopy, and frozen for histochemistry, biochemistry, and genetic studies, if necessary. To avoid post-operative hematoma, the sutured wound is compressed by a bandage for 2 hours, and the patient avoids standing up for 2 days. 

## Results and discussion 

From more than 35-year experience of combined nerve and muscle biopsy, we are convinced that this procedure significantly improves the diagnosis of systemic diseases with peripheral nerve involvement [11, 12, 13, 14, 15, 16, 17, 18, 19]. Among these diseases, vasculitides are certainly the most frequently diagnosed on neuro-muscular biopsies, but this procedure is also well advised to assess the diagnosis of sarcoidosis or amyloidosis. More occasionally, combined nerve and muscle biopsy leads to a diagnosis of cholesterol embolism, intra-vascular lymphoma, mitochondrial cytopathy, or storage disease. 

**Figure 2:**
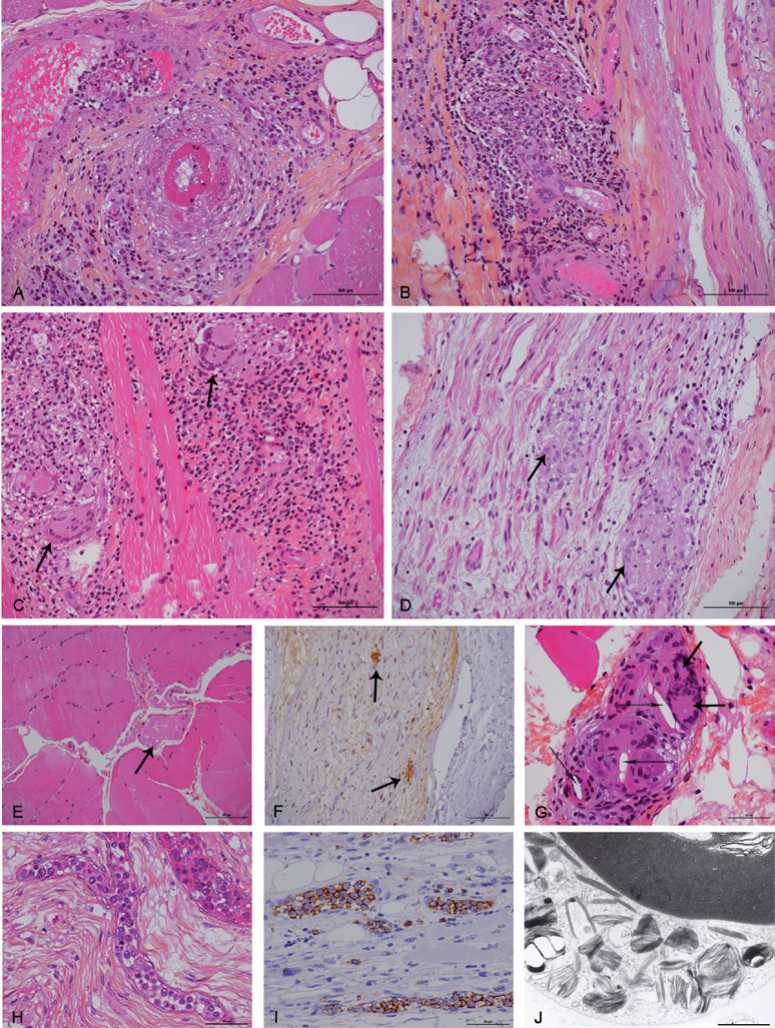
A – I: Histopathology on paraffin sections from combined nerve and muscle biopsies in neuropathic patients: hematoxylin and eosin (H & E) staining showing necrotizing vasculitis in muscle (A) and marked inflammatory infiltrates involving epineurial vessels (B); H & E staining showing well developed sarcoid granulomas with multi-nucleated giant cells (arrows) between muscle fibers (C) and smaller sarcoid granulomas (arrows) mainly composed of epithelioid cells within the endoneurium (D); H & E staining showing an amorphous amyloid deposit (arrow) between muscle fibers (E), and anti-transthyretin immuno-staining revealing two small amyloid deposits (arrows) in the endoneurium (F); H & E staining showing cholesterol embolisms (thin arrows) and multinucleated giant cells (thick arrows) within small arteries on muscle biopsy (G); H & E staining (H) and anti-CD20 immuno-staining (I) revealing intra-vascular lymphomatous cells in endoneurial vessels (H) and in muscle vessels (I). J: Electron microscopy showing numerous Pi granules within the Schwann cell cytoplasm of a myelinated fiber. Bar = 100 µm in A – F; Bar = 50 µm in G – I; Bar = 1 µm in J.

Names and definitions for vasculitides adopted by the 2012 international Chapel Hill consensus conference on the nomenclature of vasculitides are based on the type and size of involved vessels, organ involvement, underlying systemic disease, or probable specific etiology [20]. More specifically, attempts have been made on the classification of vasculitic neuropathies and on diagnostic criteria for pathologically definite, probable and possible vasculitic neuropathy [21, 22]. One characteristic histological lesion consisting in vessel wall infiltration by inflammatory cells, with or without visible parietal fibrinoid necrosis (Figure 2A, B), is mandatory to confirm the diagnosis of vasculitis, and muscle biopsy in addition to nerve biopsy increases the number of medium-sized arterioles and/or small vessels for examination [16, 23, 24, 25]. Other vascular lesions may coexist with the characteristic vascular wall infiltration by inflammatory cells with or without parietal necrosis, or may be suggestive but not diagnostic if isolated. These are acute or chronic thrombosis with recanalization, perivascular erythrocytes or hemosiderin deposits, fragmentation of internal elastic lamina, cicatricial vessel wall fibrosis, clustering of neo-vessels, and unspecific inflammatory cell infiltrates surrounding small vessels. In a series of 100 patients with necrotizing vasculitis, the muscle biopsy was more frequently positive for vasculitis (80%) than was nerve biopsy (55%) [23]. In a larger series of 202 cases, available muscle specimens improved the yield of definite vasculitis by 31.6% in necrotizing vasculitis [16]. This improvement of positive results has also been observed in other series [26, 27, 28, 29]. However, in another study when the vastus lateralis muscle was taken as well as the sural nerve, the yield of positive results was not significantly increased [30]. In Churg-Strauss syndrome, systemic vasculitis is associated with eosinophilia and adult-onset asthma. In the lungs, heart, peripheral nerves, and muscles, eosinophils are numerous within vascular cellular infiltrates and within extra-vascular granulomas [15, 31]. Non-systemic vasculitic neuropathy was first described in 20 patients [32] and then in two other series [33, 34]. In fact, necrotizing vasculitis in muscle and nerve, without visceral involvement, had already been described in 3 autopsy cases [35]. In 1 of 3 other patients with neuropathy and localized necrotizing vasculitis, vascular lesions were only visible in muscle specimens [36]. 

Sarcoid neuropathy is diagnosed histologically by identifying at least one non-caseating granuloma composed of epithelioid cells with a few multinucleated giant cells and lymphocytes [17, 37, 38, 39]. In a review of 38 sarcoid neuropathy cases, characteristic sarcoid granulomas were present in nerve in 11 cases and in muscle alone in 5. Both muscle and nerve were infiltrated in 10, nerve and other tissue in 4, and other tissue in 8 [17]. Non-caseating granulomas are well developed between muscle fibers (Figure 2C), whereas they are frequently smaller within epi- or endoneurium (Figure 2D). In addition, evidence of granulomas in muscle is decisive to differentiate sarcoidosis from tuberculoid leprosy in which muscle is always spared [38]. 

Nowadays, most cases of familial amyloid polyneuropathy are diagnosed by molecular genetic analysis on the transthyretin (TTR) gene on blood specimens [40]. Nonetheless, it is not uncommon to establish the diagnosis on nerve or muscle biopsy, and several authors favored a combined biopsy of muscle and nerve for this purpose (Figure 2E, F) [3, 14, 18, 41]. When present, endoneurial amyloid deposits are visible after hematoxylin-eosin (H & E) staining in the form of patchy acellular eosinophilic areas. Amyloid deposits are easily overlooked on muscle specimens stained with H & E, but Congo-red staining gives to these deposits an “amber” color and a characteristic “apple-green” birefringence when observed under polarizing light. Ultrastructural examination may give a decisive clue when disclosing bundles of straight fibrils, 8 nm in diameter, in a loose or tightly matted collection. Some characteristic fibrils may be in close relationship with a macrophage and correspond to very small amyloid deposits not even visible on semi-thin sections [18]. Once amyloid deposits are identified, their origin has to be specified by immunohistochemistry, even if a monoclonal gammopathy is present in the serum and/or urine. Amyloid deposits can be studied on deparaffinized sections of muscle and nerve specimens using anti-transthyretin (TTR) antibody, and a robust immuno-staining is mandatory to assess a positive result. This TTR immuno-staining can be observed around and within vessel walls, and between nerve (Figure 2F) or muscle fibers. It may reveal small amyloid deposits encasing an isolated muscle fiber or surrounding adjacent fat cells [18]. A few cases of hereditary amyloidosis due to gelsolin have been reported mainly in Finnish patients, and amyloid deposits were observed in both muscle and nerve specimens [42]. Other amyloid deposits are derived from a light chain, and the contribution of muscle biopsy had already been emphasized [43]. However, we have to be cautious because hereditary amyloidosis may be misdiagnosed in some patients having a monoclonal light chain [44, 45]. When responsible for amyloid deposits, the light chain may be isolated or associated with an IgA, IgG, or IgM monoclonal gammopathy. The light chain amyloid component is better identified by immunofluorescence on frozen nerve and/or muscle biopsy specimens, and electron microscopy differentiates the granular non-amyloid light chain deposits from the fibrillar amyloid deposits [46]. Recently, the interest of mass spectrometry of laser-dissected deposits has been underlined [47]. Amyloid deposits may be more abundant in muscle than in nerve specimens in amyloid neuropathies due to *TTR* mutations [48] as well as in cases due to λ or κ light chain [49]. 

Cholesterol embolisms may be responsible for ischemic neuropathy, but only few observations on nerve biopsy have been reported whereas their presence in muscle vessels is easier to disclose [50]. Cholesterol embolisms present as angular clear material occluding the lumen of small arterioles, often with multinucleated giant cells in close contact (Figure 2G). 

Unpredicted diagnosis of intra-vascular lymphoma may be established when numerous malignant B lymphocytes are visible within the lumen of several small vessels on nerve and muscle specimens (Figure 2H, I) [12, 51, 52]. Other lymphomas may infiltrate peripheral nerve [53, 54], but infiltration of muscle is occasional [55]. Meningoradicular involvement by lymphoma generally does not concern peripheral nerve nor muscle [56]. 

Additional muscle specimens are decisive in the diagnosis of neuropathies related to mitochondrial cytopathies [13, 19, 57]. Typical modifications can be observed on muscle frozen sections after Gomori trichrome staining, with ragged red fibers corresponding to sub-sarcolemmal accumulation of abnormal mitochondria. This is confirmed by histochemical reactions for the mitochondrial enzymes succinate dehydrogenase and cytochrome c oxidase [58]. It must be noticed that such characteristic modifications are often absent in children as well as in a few adult cases. At ultrastructural examination of muscle specimens, there are abnormal mitochondria presenting as rectangular crystalloid inclusions with a geometric lattice appearance, more often with a sub-sarcolemmal location. In addition, mitochondrial DNA (mtDNA) is better analyzed on frozen muscle specimens than on lymphocytes from circulating blood. There are a few cases of mitochondrial neuropathies due to a mtDNA point mutation responsible for MELAS (mitochondrial encephalopathy, lactic acidosis, stroke-like episodes) or MERRF (myoclonic epilepsy with ragged red fibers) phenotypes, but most cases are secondary to nuclear mutations with ensuing mitochondrial dysfunction, and usually associated with multiple small deletions of mtDNA. Most of these cases are observed in adult patients with an autosomal dominant or autosomal recessive inheritance, but a few cases are sporadic. Progressive external ophthalmoplegia is present is some cases, sometimes associated with sensory ataxic polyneuropathy. Ultrastructural examination of nerve specimens can show intra-axonal mitochondria with distorted cristae, but more demonstrative are enlarged mitochondria with abnormal cristae within the Schwann cell cytoplasm of a few myelinated fibers [13, 19]. 

Storage diseases have mainly been studied on isolated nerve biopsies [1, 2, 3, 4, 5] with characteristic features of storage material at ultrastructural examination, but the concomitant study of muscle specimens proved to be very useful in some cases [2, 11]. Pi granules or Reich granules, which are present in the Schwann cell cytoplasm of myelinated fibers, may be confused with storage material because they appear as membrane-bound inclusions containing lamellar structures displaying alternate dark and light bands with sometimes a parallel array at higher magnification (Figure 2J). Other inclusions may be present within the Schwann cell cytoplasm and likely correspond to remnant of a destroyed myelinated fiber. Such inclusions present a polymorphic content or more rarely appear as an elongated rod-like structure with a fine periodicity. They deserve to be cautiously examined under electron microscope [1, 2, 3, 4, 5] and their absence in muscle specimens is a reliable data to rule out a diagnosis of storage disease, especially Fabry’s disease [11]. 

## Conclusion 

Combined nerve and muscle biopsy, via a single skin incision, has to be performed in an appropriate surgical area, by a competent staff, with a perfect methodology and in aseptic conditions. Availability of nerve and muscle specimens for light and electron microscopy, histochemistry, biochemistry, and genetic studies, improves the diagnosis of several systemic diseases responsible for peripheral neuropathy. 

## Conflict of interest 

No conflict of interest to declare.
